# Outbreak of Tularemia in a Group of Hunters in Germany in 2018—Kinetics of Antibody and Cytokine Responses

**DOI:** 10.3390/microorganisms8111645

**Published:** 2020-10-23

**Authors:** Daniela Jacob, Anne Barduhn, Dennis Tappe, Jessica Rauch, Klaus Heuner, Daniela Hierhammer, Katharina vom Berge, Julia M. Riehm, Matthias Hanczaruk, Stefanie Böhm, Merle M. Böhmer, Regina Konrad, Berit Bouschery, Marc Dauer, Elisabeth Schichtl, Hamid Hossain, Roland Grunow

**Affiliations:** 1 Robert Koch Institute, 13353 Berlin, Germany; JacobD@rki.de (D.J.); BarduhnA@rki.de (A.B.); HeunerK@rki.de (K.H.); 2 Bernhard Nocht Institute for Tropical Medicine, 20359 Hamburg, Germany; tappe@bnitm.de (D.T.); rauch@bnitm.de (J.R.); 3 Local Health Authority, 92421 Schwandorf, Bavaria, Germany; Daniela.Hierhammer@landkreis-schwandorf.de (D.H.); Katharina.vomBerge@Landkreis-Schwandorf.de (K.v.B.); 4 Bavarian Health and Food Safety Authority, 85764 Oberschleißheim, Germany; Julia.Riehm@lgl.bayern.de (J.M.R.); Matthias.Hanczaruk@lgl.bayern.de (M.H.); Stefanie.Boehm@lgl.bayern.de (S.B.); merle.boehmer@lgl.bayern.de (M.M.B.); Regina.Konrad@lgl.bayern.de (R.K.); 5 Postgraduate Training for Applied Epidemiology (PAE), Robert Koch Institute, 13353 Berlin, Germany; 6 European Programme for Intervention Epidemiology Training (EPIET), European Centre for Disease Prevention and Control, 169 73 Solna, Sweden; 7 Institute of Social Medicine and Health Systems Research, Otto-von-Guericke-University, 39106 Magdeburg, Germany; 8 Department of Medicine II, Klinikum St. Marien, 92224 Amberg, Germany; bouschery.berit@klinikum-amberg.de (B.B.); dauer.marc@klinikum-amberg.de (M.D.); 9 Department of Medicine II, Saarland University, 66421 Homburg, Germany; 10 Kleintierpraxis im Alten Forstamt, 92536 Pfreimd, Germany; info@kleintierpraxis-pfreimd.de; 11 Institute of Laboratory Medicine and Microbiology, Klinikum St. Marien, 92224 Amberg, Germany; hossain.hamid@klinikum-amberg.de; 12 Institute of Laboratory Medicine and Microbiology, Kliniken Nordoberpfalz AG, 92637 Weiden, Germany

**Keywords:** bacterial infections, tularemia, hares, *Francisella*, *Francisella tularensis*, outbreak, antibodies, cytokines

## Abstract

In November 2018, an outbreak of tularemia occurred among hare hunters in Bavaria, Germany. At least one infected hare was confirmed as the source of infection. A number of hunting dogs showed elevated antibody titers to *Francisella tularensis*, but the absence of titer increases in subsequent samples did not point to acute infections in dogs. Altogether, 12 persons associated with this hare hunt could be diagnosed with acute tularemia by detection of specific antibodies. In nine patients, the antibody and cytokine responses could be monitored over time. Eight out of these nine patients had developed detectable antibodies three weeks after exposure; in one individual the antibody response was delayed. All patients showed an increase in various cytokines and chemokines with a peak for most mediators in the first week after exposure. Cytokine levels showed individual variations, with high and low responders. The kinetics of seroconversion has implications on serological diagnoses of tularemia.

## 1. Introduction

*Francisella tularensis* is the causative agent of the zoonosis tularemia. The bacteria infect a broad range of animals and can be transmitted to humans by different routes: direct contact with infected or contaminated animals (ulceroglandular form), alimentary ingestion (oropharyngeal form), inhalation (respiratory form), or by smear infection (oculoglandular form) [1,2] (WHO Guidelines on Tularemia, 2007 (http://apps.who.int/iris/bitstream/10665/43793/1/9789241547376_eng.pdf)). Initial clinical symptoms are often fever and enlarged lymph nodes, followed by more specific signs depending on the route of infection. Hare hunters in endemic regions for tularemia are at special risk of the disease, typically due to underestimation or lack of awareness. In countries with a low prevalence of the disease, such as Germany [3], physicians might consider tularemia as a differential diagnosis quite late in the course of the disease, leading to a delay in specific diagnostics and treatment [4,5,6]. 

In November 2018, an outbreak of tularemia occurred among hare hunters in Bavaria, Germany. At least, 42 persons and 11 hunting dogs came into contact with one or more infected hares or were indirectly involved in the hunting event. Altogether, 12/41 tested persons associated to at least one infected hare could be confirmed serologically as having acquired tularemia infection. 

In addition, the cytokine response was determined in these patients. The epidemiological analyses with determination of risk factors were conducted elsewhere [7]. Hunters who were directly involved in the processing of hares were more at risk and were 10 times more likely to be infected with *F. tularensis* than hunting participants who were not directly involved. Furthermore, hunting dogs involved were tested for specific antibodies to investigate transmission of the pathogen from dog to human that was suspected in at least one case. Here, we describe the laboratory investigation of this outbreak, facilitating the early laboratory confirmation of clinical diagnoses of tularemia in patients with early and successful antibiotic treatment. 

## 2. Methods and Materials

### 2.1. Hare Material 

The Bavarian Health and Food Safety Authority (LGL, Oberschleissheim, Germany) conducted the collection and primary investigation of the hare samples. Four out of eight hunted hares were accessible for an investigation. Organ parts were tested in the microbiology department of animal bacteriology. In addition to testing for *Francisella tularensis*, PCR was also performed for leptospires and *Brucella*. DNA from tissue was extracted with the MagNA Pure 24 system (Roche, Mannheim, Germany) as recommended by the manufacturer. For real-time PCR, the LightMix Kit *Francisella tularensis* 16S (Tib Molbiol, Berlin, Germany) and LightCycler Fast Start Set Hybridization Probes (Roche) were used. The subspecies was determined by RD1 PCR [8] after cooking bacterial cells from isolated strains.

At the Robert Koch Institute (RKI, Berlin, Germany), 16 cultures were investigated which were grown with suspected colonies for *F. tularensis* subsp. *holarctica* on Martin Lewis agar plates (BD Diagnostics, Heidelberg, Germany) or with turbidity in brain heart infusion enriched with isovitalex inoculated from different sample materials (muscle tissue, lymph node, and bone marrow) from four hares. DNA was isolated from lymph node material (A-1299/6) and bone marrow material (A-1299/7) from one hare, and the *Francisella* isolate (A-1338) was isolated from the lymph node from the same hare. The DNA isolation was performed by using the MagNA Pure 24 system (Roche), see above. A draft genome sequence was performed from *Francisella* strain A-1338 [9].

### 2.2. Samples from Hunting Dogs

Serum from 10 hunting dogs involved in the hunting event was obtained. Nine dogs were tested twice for antibodies against the lipopolysaccharide (LPS) of *F. tularensis*. Nine throat swabs and EDTA blood samples were tested for *F. tularensis* by inoculation on culture media and by specific real-time PCR.

### 2.3. Clinical Material from Humans: Blood Cultures, Throat Swabs, Serum

Altogether, 42 persons were involved in this investigation: 35 persons participated in the hunting event, two were family members, one was a veterinary assistant, and four persons were butchery employees. Three of the latter four persons stated having had contact (e.g., touched, washed, or disassembled) to the hunted hares, while one person was not sure. However, all individuals stated the days when they had contact with the hunted hares and when the activities were carried out. A manuscript on the detailed outbreak description is in progress. All clinical samples (blood cultures, swabs, and sera) of patients admitted to the Klinikum St. Marien, Amberg, Germany, were tested for pathogens at the internal Institute of Laboratory Medicine and Microbiology, Klinikum St. Marien, and tested in parallel for *F. tularensis* and *Brucella* spp. at the Specialised Laboratory for Highly Pathogenic Bacteria (ZBS 2), RKI, Germany.

Fifty-six blood cultures, ten throat swabs, and one eye swab were tested for *F. tularensis* and *Brucella* spp. by inoculation on culture media and by specific real-time PCR. Overall, 69 human sera were analyzed for antibodies against the LPS of *F. tularensis*. A signed consensus by patients for serum donation for late diagnostic investigation was obtained. All data were collected in the framework of the curative diagnostic approaches. Thus, the responsible Bavarian Ethical Committee confirmed upon request that an additional approval was not required.

### 2.4. Cultivation of Sample Material from Humans and Dogs

All throat swabs, the eye swab, and 50 µL of blood culture sample or EDTA blood were each streaked onto CHAB agar plates (CHA (Difco, Bestbion, Cologne, Germany), 1% brain heart infusion broth, 1% proteose-peptone, 1% D-glucose, 0.5% NaCl, 0.1% L-cystine, 1.5% agar, 9% sheep blood), commercial Neisseria selective medium Plus, and chocolate agar plates (both Oxoid, Wesel, Germany) at 37 °C with 5% CO_2_ and incubated for up to 3 days. For differential diagnoses, the samples were plated onto commercial Columbia blood agar plates (Oxoid). For enrichment we used the liquid medium T described by Becker et al. [10]. 

### 2.5. Genomic DNA for Molecular Analysis

DNA extraction was performed from all throat swabs, one eye swab, and all EDTA and whole blood samples. In addition, DNA extraction was conducted out of bacterial colony material (A-1338) or lymph node material (A-1299/6) and bone marrow material (A-1299/7) from hare using the QIAGEN DNeasy Blood and Tissue kit (Qiagen, Hilden, Germany) following the manufacturer’s instructions as described recently [5]. DNA elution was performed in 100 µL of QIAGEN Elution Buffer (Qiagen). 

### 2.6. PCR Detection fopA, tul4, DD brucellosis mazG, IS711, Singleplex and Multiplex Real-Time PCRs, RD1-PCR

Multiplex real-time PCR (5′ nuclease assay, TaqMan technology) targeting *fopA* and *tul4* specific for *F. tularensis* in combination with the extraction and amplification control targeting KoMa2 were performed with oligonucleotides and probes as described recently [5]. A singleplex real-time PCR assay was performed from the clinical human sample for the detection of *c-myc* as an internal extraction control. In brief: Both real-time PCR assays were run in a total volume of 25 µL, including 5 µL of DNA samples to be analyzed. Samples were analyzed in duplicate in each run. Amplification was performed in an Applied Biosystems 7500 Real-Time PCR System (ThermoFisher Scientific, Langenselbold, Germany), each run including 40 cycles. The block PCR of the region of difference 1 (RD1-PCR) was used for the subspecies differentiation of *F. tularensis* as described recently [5]. The PCR was carried out using 15–100 ng of template DNA according to the protocol described by Broekhuijsen et al. [8]. 

The multiplex real-time PCR (5′ nuclease assay, TaqMan technology) targeting *mazG* and IS711 specific for *Brucella* spp. in combination with the extraction and amplification control targeting KoMa2 were performed with the following oligonucleotides and probes for mazG: mazG-F 5′-ggATCTgATCgTAgCgACggA-3′, mazG-R 5′-CgTCCAATgTCTCACTggAAAA-3′, Bru-mazG-TM-Multi: 5′-FAM-TgCCTTACATgggCgAACTCgAACgT-BHQ-1-3′ and for IS711: BruIS-F 5′-gCCATCAgATTgAATgCTTTTTTAAC-3′, BruIS-R 5′-AACCAgATCATAgCgCATgCg-3′; Bru-IS-TM-Multi 5′-Cy5-CgCTgCgATgCgAgAAAACATTgACC-BHQ-2-3′. The oligonucleotides and probes targeting KoMa2, the PCR assay, and conditions are described in Jacob et al. [5]. 

### 2.7. Whole Genome Sequencing

Whole genome sequencing (WGS) from DNA of sample A-1338, one *Francisella* isolate from a hare belonging to the outbreak, was performed and analyzed regarding the biovar and genetic clade of the respective strain [9]. Library pool sequencing was performed in paired-end mode on a MiSeq Instrument (Illumina, San Diego, CA, USA); for the next generation sequencing of sample A-1338-1 Illumina sequencing in combination with Nextera XT library generation was used (Illumina), as recently described [5].

### 2.8. Enzyme Linked Immunosorbent Assay (ELISA) and Western blot (WB)

An ELISA was used for screening and WB for confirmation of antibodies against *F. tularensis* LPS. Both in-house assays are accredited by DIN EN ISO/IEC 17025:2005 and DIN EN ISO 15189:2014 and have been described elsewhere [11]. Briefly, a 96-well microtiter plate Nunc-Polysorb (Thermofisher Scientific, Berlin, Germany) was coated with purified LPS from the live vaccine strain as antigen (Micromun, Greifswald, Germany). Bound human antibodies to *F. tularensis* LPS were detected by polyvalent or monovalent goat anti-human IgA, IgM, and IgG horseradish peroxidase-conjugated secondary antibody (Dianova, Hamburg, Germany) and subsequent substrate reaction. Serum dilutions starting with 1:500 that revealed an optical density above the validated cut-off were counted as positive. The dog sera were tested by the same ELISA approach but an anti-dog IgG horseradish peroxidase-conjugated secondary antibody (Dianova, Hamburg, Germany) was used (this assay was not yet fully validated, but a number of unrelated dog sera showed negative results only). For the WB, the soluble fraction of formalin-inactivated live vaccine strain was separated using sodium dodecyl sulphate polyacrylamide gel electrophoresis (SDS-PAGE) and then transferred to polyvinylidene difluoride (PVDF) ImmobilonP-Millipore membranes (Roth, Karlsruhe, Germany). Using polyvalent horseradish peroxidase-conjugated secondary antibodies, the typical LPS ladder revealed the presence of specific anti-*F. tularensis* antibodies. The final results were obtained after confirmation of the ELISA results by WB: “positive” denoted strong bands, “negative” almost no bands, and “borderline” weak but clearly visible bands.

### 2.9. Cytokine and Chemokine Measurements

Serum cytokine and chemokine responses were analyzed by LegendPlex assay (BioLegend, Fell, Germany). See Supplementary Table S1 for the analyzed mediators and their respective detection limits. For time sampling points 1–3 (1, 2, and 3 weeks after exposure), sera from all nine patients for whom antibody testing was performed were available. For time sampling point 4 (21 weeks after exposure), only sera from patients 1, 3, 5, 6, 7, and 9 were available. As controls, 16 sera from healthy blood donors who were seronegative for tularemia were employed. These 16 sera were randomly picked from a larger group of negative sera. Patients were grouped according to their respective time sampling points. Statistical analysis was performed with GraphPad Prism 7 software (GraphPad Software Inc., La Jolla, CA, USA). For comparison between the analyzed groups, the Kruskal–Wallis test with subsequent Dunn’s multiple comparisons test was used.

## 3. Results

### 3.1. Pathogen Detection in Hares, Humans, and Hunting Dogs

At the Institute of Laboratory Medicine and Microbiology in Amberg, all blood cultures of patients admitted to the hospital remained negative until day 10 of incubation. In the framework of the initial laboratory diagnostic attempts, all throat samples were negative for Influenza A/B and Respiratory Syncytial Virus (RSV). Cultivation of throat swabs showed growth of normal bacterial flora of the oral cavity but no evidence of pathogenic bacteria or fungi. All sera were tested negative for *Leptospira* IgM and IgG.

At the Robert Koch Institute, one hare out of four investigated animals was confirmed to be infected with *F. tularensis* subsp. *holarctica* by PCR detection. DNA from *Francisella* strain (A-1338) isolated from this hare and DNA isolated from the lymph node of the same hare (A-1299/6) has been sequenced, and phylogenetic analysis indicated that the *F. tularensis* ssp. *holarctica* strain belonged to biovar II, clade B.12 and subclade B.33 [9]. All throat swabs and blood cultures from the patients investigated one week after exposure remained negative in PCR testing for *F. tularensis* and *Brucella* spp. Neither subsequent cultures from blood cultures and throat swabs nor the eye swab showed growth of these bacteria. 

All investigated samples from hunting dogs remained negative for *F. tularensis* in both culture approaches and PCR. 

### 3.2. Antibody Detection in Hunting Dogs

Ten of the 11 dogs involved were sampled close to the beginning of the outbreak. Several dog sera were positive but did not show dynamics for acute or new infections when testing paired sera within 8 days (Table 1).

### 3.3. Serology and Antibody Kinetics in Humans

As all blood cultures and throat swabs remained negative in PCR and culture, serological confirmation was the only approach to confirm an infection with *F. tularensis*. Altogether, 12/41 persons related to this outbreak could be serologically confirmed by ELISA and WB (data on WB not shown). In total, 10/35 hunting participants and 2/4 butchery employees were tested positive. Because tularemia was early suspected, the development of antibodies could be monitored in nine affected hunters, starting one week after exposure to confirm the clinical diagnoses with laboratory methods (Table 2). 

**Figure 1:**
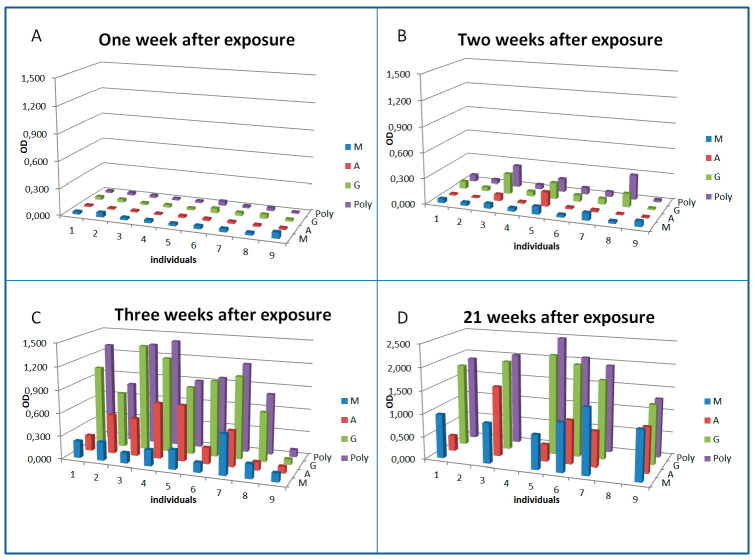
Seroconversion and dynamics of antibody isotypes against *F. tularensis* in patients treated early with antibiotics. Sera from nine patients diagnosed with acute tularemia were analyzed by ELISA. For sampling time points 1–3 ((**A**) sampled on 4th November 2018, one week after exposure; (**B**) sampled on 8th November 2018, two weeks after exposure; (**C**) sampled on 14th November 2018, threeweeks after exposure), sera from all nine patients were available. For sampling time point 4 ((**D**) sampled in April/May 2019, 21 weeks after exposure), only sera from six patients were available. Exposure date was 27th October 2018 and patients were admitted with clinical signs to the hospital on 1st November 2018. The validated diagnostic cut-offs of this assay are: positive OD > 0.6, borderline OD 0.25–0.6, negative < 0.25.

All nine patients were negative for antibodies against the pathogen one week after exposure (Figure 1A–D). 

Thus, all subsequent detection of antibodies could be taken as confirmation of the diagnosis. It can be seen that some patients started to develop specific antibodies one week after exposure and in parallel to the development of clinical symptoms. Interestingly, the patients reacting earliest (patients 3, 5, and 8) showed a predominant IgG response combined with IgA (3 and 5) rather than an IgM response at that early stage of the infection. The overall antibody response detected by a polyvalent anti-Ig conjugate was mainly determined by the IgG response. Three weeks after exposure and two weeks after clinical onset of the disease, all but one patient had developed specific antibodies. In one exposed patient [9], antibodies could not be detected during three weeks after exposure, but 21 weeks after the infection, when we were able to obtain another blood sample to check for seroconversion again, antibodies were detected.

### 3.4. Cytokine and Chemokine Changes During Infection

**Figure 2:**
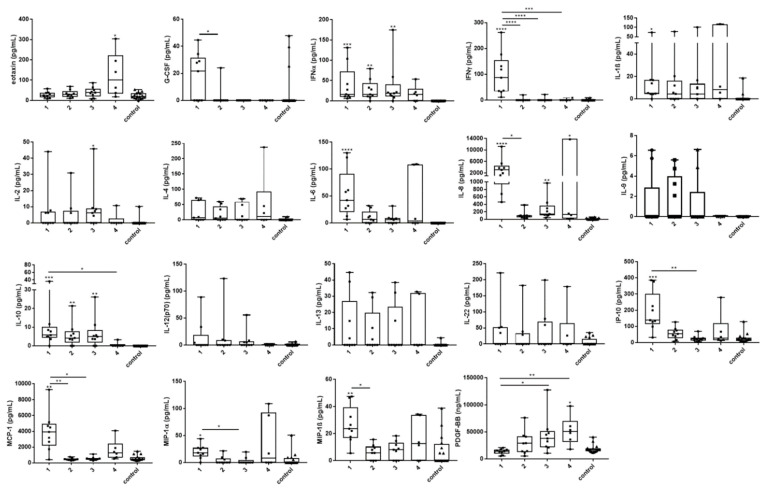
Changes of serum cytokine and chemokine levels in acute tularemia cases over time. Sera from nine patients diagnosed with acute tularemia and 16 sera from healthy blood donors (controls) were analyzed by bead-based LegendPlex assay. Time sampling points are shown on the X axis (1 – 3 represent 1, 2, and 3 weeks after exposure; 4 represents 21 weeks after exposure). For sampling points 1–3, sera from all nine patients for whom antibody testing was performed were available. For sampling time point 4, only sera from six patients were available. In comparison to the healthy controls and/or between different time points, significant changes of eotaxin, G-CSF, IFNα, IFNγ, IL-1β, IL-2, IL-6, IL-8, IL-10, IP-10, MCP-1, MIP-1α, MIP-1β, and PDGF-BB were demonstrated in the tularemia patients. Non-significant but marked changes were seen for IL-4, IL-9, IL-12, IL-13, and IL-22. Many mediators peaked at the first sampling time point, whereas peaks at sampling time point 4 were caused by patients 3, 6, and 7. Patients 4 and 7 showed the highest cytokine and chemokine responses throughout. For comparison between the analyzed groups, the Kruskal–Wallis test with subsequent Dunn’s multiple comparisons test was used. Data are expressed as box-and-whisker plots showing median, upper and lower quartile, minimum, and maximum values. Asterisks indicate statistically significant differences (* *p* < 0.05, ** *p* < 0.01, *** *p* < 0.001, **** *p* < 0.0001). Asterisks with bars represent comparisons between the respective time sampling points. Asterisks without bars represent the comparison of individual time sampling points versus the control group. Serum concentrations of FGF-basic, GM-CSF, IL-5, IL-17A, IL-17F, IL-21, RANTES, TNFα, and VEGF were similar in the patients and the controls. Generally, most cytokine and chemokine levels peaked in the first week after exposure, followed by normalization over the next weeks. Of note, some cytokines and chemokines peaked at blood sampling time point 4 instead (eotaxin, IL-4, PDGF), or showed a second peak at time point 4 (IL-1β, IP-10, MCP-1, MIP-1α, MIP-1β). This second peak seen in the patient cohort 21 weeks after exposure was mainly due to elevations of these cytokines in patients 3, 6, and 7. Patients 4 and 7 showed the highest concentrations for most cytokines and chemokines tested at the first three sampling points (high responders). In contrast, patient 9 who seroconverted later than three weeks post exposure showed generally the lowest cytokine and chemokine concentrations throughout the observation period (low responder).

Serum levels of eotaxin, G-CSF, IFNα, IFNγ, IL-1β, IL-2, IL-6, IL-8, IL-10, IP-10, MCP-1, MIP-1α, MIP-1β, and PDGF were significantly increased in tularemia patients when compared to healthy controls and/or between different time points of sampling. Marked changes, however without statistical significance, were found for IL-4, IL-9, IL-12, IL-13, and IL-22 (Figure 2).

## 4. Discussion

With regard to tularemia in humans, Germany represents a region of low incidence. *F. tularensis* subsp. *holarctica* is endemic and the only species known so far to cause tularemia in Germany [3,11,12,13]. Up to 50 cases of tularemia are reported per year but the number increases, indicating that tularemia is a re-emerging disease [3,14]. Studies on *Francisella* isolates from humans and wild animals revealed a high genetic diversity of *F. tularensis* subsp. *holarctica* in Germany [9,12,13,15]. Phylogenetic analysis demonstrated that *F. tularensis* subsp. *holarctica* of biovar I (erythromycin-susceptible isolates) are mainly found in Western Europe and isolates of biovar II (erythromycin-resistant strains) occur in Northern and Eastern Europe. A similar North–West pattern is seen in Germany [1,9,16,17,18,19,20].

In Germany, most human isolates belong to the phylogenetic clade B.12 (biovar II) or clade B.6 (biovar I). However, it seems that isolates of clade B.6 are more often isolated from tularemia patients with pneumonia than from individuals with other forms of the disease [9]. Biovar I (clade B.6) is more commonly found in human isolates from Bavaria [9]. In the outbreak described here, a *Francisella* isolate belonging to clade B.12/B.33 could be isolated from one of the hunted hares. The same was true for an uncommon tularemia outbreak associated with contaminated fresh grape must, which revealed *F. tularensis* subsp. *holarctica* belonging to the B.12 (B.34) phylogenetic clade as the causative agent. This outbreak occurred in Rhineland-Palatinate and involved six cases. Generally, strains of biovar I are dominant in this region [4,5,9].

It was shown that infected or contaminated dogs can infect humans [21]. One of our patients was not involved in the processing of hunted hares but had only contact to participating hunting dogs that were fed the remains of the processed hares. The serological investigation showed that probably 4/10 dogs had been exposed to *F. tularensis* prior to the outbreak described here, indicating a high prevalence of the pathogen in wildlife of this region. Dogs that tested positive did not show an increase in the antibody titer in paired sera taken with a time shift of eight days. However, it is not excluded that contaminated or infected dogs contributed to the human infections in this outbreak. Further investigation of hunting dogs with well validated assays is required to confirm this conclusion.

The diagnosis of tularemia is often delayed due to the low prevalence of this zoonosis and the lack of awareness. After starting treatment with effective antibiotics like ciprofloxacin, it can be difficult to detect the pathogen. Recently, we have seen an increased detection rate conducting long-term blood cultures (up to 10 days) only when blood was taken before antibiotic treatment of the patients. Therefore, the diagnosis is often based on antibody detection and highly specific and sensitive assays are available [11,22].

In this outbreak, tularemia was suspected already one week after the exposure when the very first clinical flu-like symptoms like fever and malaise became apparent. This was very unusual and was based on the awareness of one of the hunters. The affected persons had admitted themselves to the hospital and, based on the patients’ history and clinical symptoms, the treatment with first choice antibiotics (ciprofloxacin 2 × 400 mg intravenously. or 2 × 500 mg orally) according to the recommendation of the Robert Koch Institute (https://www.rki.de/DE/Content/Kommissionen/Stakob/Stellungnahmen/Stellungnahme_Tularaemie.pdf?__blob=publicationFile (in German language)) [23] had been started immediately without laboratory confirmation of tularemia. However, the laboratory diagnostics started at the same time in parallel. As no visible manifestations of the disease such as skin ulcera or swollen lymph nodes were present at that time, blood cultures and throat swabs for detection of the pathogen as well as serum for detection of specific antibodies were the only clinical samples with the potential to confirm the disease. All samples taken from the patients remained negative for *Francisella* cultivation and genome detection as well as for other pathogens. Due to the effective antibiotic treatment, the patients recovered quickly and were discharged after one week while continuing the antibiotic treatment at home. In spite of the obvious epidemiological context and in order to verify the correct antibiotic treatment, appropriate measures were undertaken to confirm the clinical diagnosis by laboratory evidence.

Sera taken one week after exposure were also tested negative for specific anti-*Francisella*-LPS antibodies. Thus, RKI’s laboratory was requested by the treating physicians to confirm the clinical diagnosis as early as possible. Sera were repeatedly investigated to confirm or exclude tularemia. Eight out of nine clearly exposed individuals who all took part in the handling of the hunted hares developed antibodies three weeks after exposure (two weeks after onset of the disease). Interestingly, the diagnosis of tularemia in one patient could be confirmed only with the sample taken 21 weeks after exposure. This patient had no relevant previous illnesses and no home medication. He presented clinically with cephalgia and small wounds on his hands and small painful lymph nodes in the left axilla and on his upper arm. In the first blood count, he had a subtle bicytopenia (thrombopenia and leukopenia). We hypothesize that this could be due to a low bacterial load or due to a delayed or impaired immune response as reflected by the comparably low cytokine and chemokine levels in this patient. The patient did not report other risks of exposure until the detected seroconversion. Our data show that all exposed and hospitalized individuals became clinically ill and developed specific antibodies against the pathogen. The dynamics of antibody formation is individually different and the time span to develop antibodies detectable by standard ELISA might be longer than three weeks. There was no dominant immunoglobulin isotype involved. This confirms earlier observations that in tularemia not only IgM indicates an acute infection, but that rather all isotypes might be elevated at an early stage of infection [24]. It can be assumed that specific antibodies can be detected earlier when using lower initial dilutions of the serum (in our assay 1:500 was evaluated for best specificity and sensitivity), but in this case any lower specificity of the assay must be considered accordingly.

In our study, we tested antibody kinetics in parallel with cytokine kinetics. While antibodies were detectable three weeks after the exposure to the pathogen, significant changes of cytokine and chemokine levels already occurred in the first week after the exposure. The highest elevations for most mediators in our patients were seen in week 1 after exposure, with normalization of most cytokines and chemokines during the following weeks. Of note, the patients received antibacterial therapy from the eighth day after the hunt (day of inpatient admission) which might have influenced the cytokine and chemokine concentrations afterwards. Some of our patients showed high cytokine and chemokine responses in general, whereas others were classified as low responders. The differences in the inflammatory response may indicate an individual feature or may depend on the bacterial load (infective dose). It remains unclear whether the late cytokine response (second peak) of patients, especially of individuals 3, 6, and 7, was still caused by the tularemia infection or by other effects. It could be of interest to study long-term kinetics of cytokines in tularemia patients, as prolonged courses of tularemia infection have been described before, while the pathogenesis in these cases is unclear [25,26].

In our patients, a hypercytokinemia was observed which is generally regarded to be characteristic of the immune dysregulation during an acute infection. Mixed Th1 (IFNγ, IL-2, IL-12) and Th2 (IL-4, IL-13) responses were seen with elevations of both pro-inflammatory and anti-inflammatory cytokines as well as regulatory cytokines and chemokines. The role of cytokines and chemokines in host responses during tularemia is only beginning to be elucidated [27]. There are several animal models, but only few studies involving human subjects are described. The rapid production of pro-inflammatory and Th1-type cytokines, especially IFNγ, is critical for the initial control of *Francisella* infection; however, little is known about the role of Th2 cytokines [27]. Chemokines, such as IL-8, IP-10, MCP-1, MIP-1α, and MIP-1β, were found to be upregulated during the acute phase of infection in our study; they are known to attract neutrophils and macrophages into inflamed tissue, promoting leukocyte–endothelial cell interaction. The elevation of eotaxin and PDGF might possibly reflect convalescence, as these concentration peaks were seen at time sampling point 4.

Previous in vitro studies with human monocytes and monocyte-derived macrophages infected with the *F. tularensis* live vaccine strain (LVS) demonstrated elevated concentrations of G-CSF, IFNγ, IL-1β, IL-2, IL-6, IL-8, IL-10, IL-12p70, IL-17, IP-10, MIP-1α, MIP-1β, TNFα, and VEGF in the cell culture supernatants [28,29,30]. Here, we demonstrate very similar changes over time during a natural infection, an observation that has—to the best of our knowledge—so far only been made longitudinally after vaccination of human subjects with the LVS [31]. In the vaccination study, elevations of plasma levels of FGFb, IFNγ, IL-4, IP-10, MIP-1β, RANTES, and PDGF were described, with highest levels on day 1 and 2 (out of days 1, 2, 7, and 14) post vaccination [31].

## 5. Conclusions

Seroconversion occurred after individual time intervals within a time frame of three weeks. Serological diagnosis of tularemia could be confirmed most reliably aroundthree weeks after exposure and two weeks after onset of clinical symptoms. However, the development of antibodies may be considerably delayed in some individuals, and multiple serial blood samples have to be analyzed in order to diagnose tularemia when the disease is clinically suspected. Thus, although *Francisella tularensis* was not directly detectable in patients due to the early administration of antibiotics, the clinical signs and epidemiological context allowed us to suspect an infection with *F. tularensis*. The seroconversion within three weeks after exposure in most of the patients confirmed an infection with *F. tularensis*.

## Figures and Tables

**Table 1:** Detection of IgG antibodies by ELISA in sera from hunting dogs.

Dog No/Sample Identification	Serum 1 (Day 1)		Serum 2 (Day 8)	
	OD	Result	OD	Result
1/A-1278	0.021	negative	0.026	negative
2/A-1279	0.017	negative	0.018	negative
3/A-1280	1.581	strongly positive	1.409	strongly positive
4/A-1281	0.013	negative	0.035	negative
5/A-1282	0.156	weakly positive	0.121	weakly positive
6/A-1283	1.479	strongly positive	1.839	strongly positive
7/A-1284	0.448	weakly positive	0.382	weakly positive
8/A-1285	0.014	negative	0.018	negative
9/A-1286	0.022	negative	0.030	negative
10/A-1301	0.026	negative	Not tested	Not tested

**Table 2:** Timeline of activities.

Index	Date	Action	Time After Exposure (Day/Weeks)	Time After Clinical Signs (Day/Weeks)
0	27 October 2018	Exposure	0	0
1	01 November 2018	Flu-like clinical signs, hospitalization	5/1	0
2	04 November 2018	First set of sera	8/1	3/0
3	08 November 2018	Second set of sera, discharge of patients from hospital in good health conditions	12/2	7/1
4	14 November 2018	Third set of sera	18/3	13/2
5	April/May 2019	Fourth set of sera	Approx. 150/21	Approx. 150/21

## Supplementary Materials

The following are available online at https://www.mdpi.com/2076-2607/8/11/1645/s1. Supplementary Table S1. Cytokines and chemokines measured in the study.
