# Supplementary material for: Outbreak of Tularemia in a Group of Hunters in Germany in 2018—Kinetics of Antibody and Cytokine Responses
Source: Microorganisms. 2020 Oct 23;8(11):1645. doi: 10.3390/microorganisms8111645 (PMC7690809; doi:10.3390/microorganisms8111645)
Supplement: Supplementary file 1 [file microorganisms-08-01645-s001.pdf]

Supplementary Table S1. Cytokines and chemokines measured in the study.

Mediator	Abbreviation	Detection Limit (pg/mL)
eotaxin	N/A	4.7
fibroblast growth factor basic	FGFb	24.2
granulocyte colony-stimulating factor	G-CSF	19.6
granulocyte-macrophage colony stimulating factor	GM-CSF	6.7
interferon-α	IFNα	5.6
interferon-γ	IFNγ	5.2
Interleukin 1β	IL-1β	4.1
Interleukin 2	IL-2	4.3
Interleukin 4	IL-4	4.8
Interleukin 5	IL-5	4.1
Interleukin 6	IL-6	4.4
Interleukin 8	IL-8	1.5
Interleukin 9	IL-9	4.0
Interleukin 10	IL-10	3.3
Interleukin 12p70	IL-12p70	4.2
Interleukin 13	IL-13	3.3
Interleukin 17A	IL-17A	3.4
Interleukin 17F	IL-17F	4.5
Interleukin 21	IL-21	6.4
Interleukin 22	IL-22	19.5
interferon-γ–induced protein-10	IP-10	N/A
monocyte chemotactic protein-1	MCP-1	N/A
macrophage inflammatory protein-1α	MIP-1α	5.0
macrophage inflammatory protein-1β	MIP-1β	5.2
platelet-derived growth factor bb	PDGF-BB	N/A
regulated on activation, normal T cell expressed and secreted	RANTES	N/A
tumor necrosis factor- α	TNFα	4.4
vascular endothelial growth factor	VEGF	27.0

N/A, not available or not applicable.
